# Quality of Life and Mental Health in Children and Adolescents after the First Year of the COVID-19 Pandemic: A Large Population-Based Survey in South Tyrol, Italy

**DOI:** 10.3390/ijerph19095220

**Published:** 2022-04-25

**Authors:** Verena Barbieri, Christian Josef Wiedermann, Anne Kaman, Michael Erhart, Giuliano Piccoliori, Barbara Plagg, Angelika Mahlknecht, Dietmar Ausserhofer, Adolf Engl, Ulrike Ravens-Sieberer

**Affiliations:** 1 Institute of General Practice and Public Health, Claudiana College of Health Professions, 39100 Bolzano, Italy; christian.wiedermann@am-mg.claudiana.bz.it (C.J.W.); giuliano.piccoliori@am-mg.claudiana.bz.it (G.P.); barbara.plagg@am-mg.claudiana.bz.it (B.P.); angelika.mahlknecht@am-mg.claudiana.bz.it (A.M.); dietmar.ausserhofer@claudiana.bz.it (D.A.); adolf.engl@am-mg.claudiana.bz.it (A.E.); 2 Department of Public Health, Medical Decision Making and Health Technology Assessment, University of Health Sciences, Medical Informatics and Technology, 6060 Hall, Austria; 3 Department of Child and Adolescent Psychiatry, Psychotherapy, and Psychosomatics, University Medical Center Hamburg-Eppendorf, 20251 Hamburg, Germany; anne.kaman@uke.de (A.K.); erhart@ash-berlin.eu (M.E.); ravens-sieberer@uke.de (U.R.-S.); 4 Alice Salomon University of Applied Sciences, 12627 Berlin, Germany; 5 Department of Health Management, Apollon University of Applied Sciences, 28359 Bremen, Germany; 6 Faculty of Education, Free University of Bolzano, 39100 Bolzano, Italy

**Keywords:** COVID-19, mental health, quality of life, anxiety, depression, children and adolescents

## Abstract

Background: Methodological heterogeneity of studies and geographical variation limit conclusions about the impact of the COVID-19 pandemic on the mental health of youth. This study aimed to explore the health-related quality of life and mental health of children and adolescents in the second year of the pandemic in South Tyrol, Italy. Methods: An online survey representative for the age and gender of the children and adolescents in South Tyrol was conducted among 5159 families with children and adolescents aged 7–19 years, between 28 May and 16 June 2021. The survey collecting parental ratings and self-rated questionnaires from children and adolescents aged 11–19 years included instruments to measure health-related quality of life (KIDSCREEN-10), mental health problems (SDQ), anxiety (SCARED), and depression (CES-DC). The results were compared with data from corresponding studies conducted in Germany. Results: Decreased health-related quality of life and increased conduct problems, peer-related mental health problems, anxiety, and depressive and psychosomatic symptoms in children and adolescents observed in the second year of the pandemic in Germany were confirmed in the second year in South Tyrol. Children and adolescents with low socioeconomic status, a migration background, and limited living space were significantly affected. Female sex and older age were associated with increased psychosocial problems and a positive family climate supported the mental health of children and adolescents during the pandemic. Conclusions: Confirmation of findings of decreased health-related quality of life and increased emotional problems after the first year of the pandemic supports the ongoing call for low-threshold health promotion, prevention, and early intervention programs to support children and adolescents who have been severely affected by the pandemic.

## 1. Introduction

As a result of school closures and restrictions due to COVID-19, children and adolescents have changed their health-related behaviors, including screen exposure, physical activity and fitness, sedentariness, sleep patterns, and eating habits related to social isolation and social deprivation [1,2]. The new reality in everyday life and education has burdened existing conditions and led to the deterioration of anxiety and mood symptoms as well as developmental, stressor-related, and eating disorders [3,4,5]. Survey results obtained in the first year of the COVID-19 pandemic globally suggest that 1 in 4 youth report clinically elevated depression symptoms, while 1 in 5 youth was experiencing clinically elevated anxiety symptoms; these mood symptom estimates are double pre-pandemic estimates [6]. Available data are short-term, and long-term harms are likely to be magnified by further school closures and social deprivation encountered during the pandemic [7].

Depression and anxiety symptoms were higher in studies conducted later in the pandemic, and in girls, depression symptoms were higher in older children [6]. Students who lived in higher-risk areas presented with severe anxiety and depression [8], especially during the late, diffusion attenuation period of the COVID-19 epidemic related to the delayed emergence and long-term persistence of psychological disorders caused by posttraumatic stress disorder [9]. The effect sizes were larger in European and Asian countries [10]. Owing to the methodological heterogeneity of the studies and the differences in the regional COVID-19 rates and measures against the pandemic, however, it is challenging to draw definitive conclusions about the real impact of the COVID-19 pandemic on the mental health of children and adolescents [11] as well as the geographical variation in that impact.

Challenges of research on child and adolescent mental health during the COVID-19 pandemic include (i) targeting selected samples (e.g., clinical populations and health workers) rather than the general population; (ii) only recruiting/reporting on non-representative samples; (iii) often focusing on a restricted set of mental health outcomes, missing the broader picture of mental and physical health, quality of life, and functioning; (iv) failing to use a longitudinal design, and; (v) collecting only parental ratings or self-rated questionnaires from children and adolescents, but not both, as summarized most recently by Solmi et al. [12].

The most important weakness of a large number of cross-sectional surveys and uncontrolled studies of acceptable quality published during the first year of the pandemic is the low representativeness of the samples [6]. Population-based, representative studies targeting the general population in a longitudinal design, covering the broader picture of psychosocial health, and collecting both parental ratings and self-rated questionnaires from children and adolescents include COPSY Germany (Impact of COVID-19 on Psychological Health), which explored the health-related quality of life (HRQoL) and mental health of children and adolescents aged 7–17 years during the COVID-19 pandemic [13,14] and compared the results to pre-pandemic data of a nationionally representative study of the behavior and well-being of children and adolescents in Germany (BELLA study) [15].

In Italy, a large European country that was hardly hit by the first wave of the pandemic with high COVID-19 incidence rates, the public schools were closed for major parts of the second half of the 2019–2020 school year; during the second COVID-19 wave, high schools were closed again, with students switching to “integrated digital learning” nationwide from November 2020 to April 2021. In South Tyrol, an autonomous province in northwest Italy, several different restrictive lockdowns were imposed to contain the pandemic.

The aim of the present study using survey items and instruments of COPSY Germany, therefore, was to perform an analogous, representative survey in South Tyrol, the alpine province of Italy with the majority of inhabitants German-speaking. Language and culture date back to the Germanic, Alemannic, and Bavarian tribes that crossed today’s South Tyrol during the Migration Period and to some extent settled there. The minority language group is Italian, which is, in cultural and historical terms, the most recent arrival and experienced its strongest growth group in the Fascist era in the 1920s and 1930s when politics tried to emphasize the “Italian character” of South Tyrol by promoting massive immigration from the south [16]. The main hypotheses for COPSY South Tyrol were that the HRQoL and mental health of children and adolescents would be impaired during the COVID-19 pandemic. Specifically, we also expected that children and adolescents would feel burdened by the pandemic, show a decrease in HRQoL, an increase in mental health problems, higher levels of anxiety, depression, and more psychosomatic symptoms during the pandemic compared to data from Germany before the pandemic. We assumed that children and adolescents with low socioeconomic status, migration background, single parenthood, and limited living space would be significantly affected. We aimed to analyze for similarities and differences in these outcomes between South Tyrol and Germany and to compare children’s and adolescents’ needs during the pandemic in South Tyrol with those of Germany. The results could guide policymakers’, health professionals’, and parents’ needs for further decisions in safeguarding the mental health of children at the regional level.

## 2. Materials and Methods

### 2.1. Study Design and Sample

In South Tyrol, public schools include elementary (*scuola primaria,* attended for 5 years), middle (*scuola secondaria di primo grado*, attended for 3 years), and high schools (*scuola secondaria di secondo grado*, attended for 5 years) [17]. Elementary schools were closed or switched to distant learning between March 2020 and May 2021, for approximately one-third of the regular operating time. In middle schools, this amount was even higher (about 40%); distance learning and school closures accounted for over 50% of the regular teaching time in high schools. The province-wide, population-based COPSY South Tyrol 2021 study was conducted as an anonymous online survey performed in early summer when schools started to open again. The online survey used the SoSci Survey Software, Version 3.2.46 (SoSci Survey GmbH, Munich, Germany).

In collaboration with the public schools’ administration, a total of about 38,400 families with children attending a public school were invited by email to participate in the online survey. Parents or guardians were contacted by the directorates of the schools the children attended. A link included in the email led to the description of the study. Informed about the study, parents were asked for their informed consent and the children’s assent. After one week, the invitation was repeated. We used a short period for data assessment to avoid the potential effects of changes in infection control measures during the pandemic.

The questionnaire used was the COPSY Germany 2020 questionnaire [13]. The study design and methodology were similar to the Germany-wide, representative COPSY Germany studies [13,14,18], which follow BELLA [15], the German health module for the examination of children’s and adolescents’ mental health. Both the COPSY Germany studies and the BELLA study provide population-based, comparable results, presenting the situation during the prior waves of the pandemic and prior to the COVID-19 pandemic, respectively.

The online questionnaire was made up of 64 questions for parents, followed by 34 questions for adolescents. Once, the parent had completed the parent’s proxy version, the questionnaire was terminated automatically for children from 7 to 10 years and went on with the adolescents’ self-report for older children. The questionnaire offered the possibility to be interrupted at any of the 98 questions and to go on later at the point of interruption. Altogether, the questionnaire took adults about 20 min and adolescents about 15 min. Participation was monitored in detail. Within the first 2 pages of the questionnaire, 18% of the parents terminated the online survey, and a further 7.8% terminated before having completed all sociodemographic questions. These questionnaires were not regarded in the analyses. 10.6% of the parents terminated before reaching the last question. 11.22% of the adolescents did not start answering the questionnaire, and 6.4% terminated before completing all sociodemographic data. These questionnaires were excluded from the analyses. A further 6.4% terminated before they reached the last question. All others completed the questionnaire. For analyses, we used all questionnaires where more than the sociodemographic variables have been answered. For comparisons between parents and adolescents, only questionnaires answered by both sides were included.

### 2.2. Measures

#### 2.2.1. Sociodemographic Variables

Youth from middle and high schools (11 to 19 years of age) responded to the self-report version of the online survey, and parents of all children and adolescents from elementary, middle, and high schools (7 to 19 years of age) answered the parent proxy version. The age and gender of children and adolescents were collected, as well as of their parents. In addition, the parent’s proxy survey included questions on marital status, migration background, occupational status, and parental education according to the “Comparative Analysis of Social Mobility in Industrial Nations” (CASMIN) index [19,20].

#### 2.2.2. COVID-19 Burden

The burden of the pandemic was explored using pandemic-focused items. Parents were asked about their perceived overall burden as well as the burden caused by pandemic-dependent changes in their work. Both parents and children were asked about pandemic-caused burdens regarding school closure, social distancing, family climate, use of digital media, changes in sports behaviors, and nutrition behaviors. The items were newly developed for the COPSY Germany 2020 questionnaire and were successfully pilot tested for feasibility, comprehensibility, and length [13,14].

#### 2.2.3. HRQoL and Mental Health

Like the COPSY 2020 Germany study, the COPSY South Tyrol 2021 study used internationally validated questionnaires following the recommendations of the International Consortium for Health Outcomes Measurement [21]. The same measures were used in the BELLA study; thus, a comparison with valid data before and during the pandemic was possible.

HRQoL was assessed using the KIDSCREEN-10 index, a valid measure of a general HRQoL factor in children and adolescents; Cronbach alpha 0.82 (0.78), test–retest reliability intraclass correlation coefficient 0.70 (0.67) for the self-(proxy-)report version [22]. The ten questions on physical, psychological, social, and school-dependent items were presented on a 5-points response scale. A sum score lying one standard deviation (8.434) below the mean (51.1846) of the BELLA study before the pandemic was categorized as “low” for adolescents’ self-reports and the standardized mean of 50 and standard deviation of 10 [23].

The strength and difficulties questionnaire (SDQ) [24,25] assesses mental health on a total problem scale and four subscales: emotional symptoms, conduct problems, hyperactivity, and peer problems. The internal consistency and validity of the SDQ total difficulties were reported for various languages and found good for most countries including Germany and Italy [26]. The parent version of the questionnaire was used. The total problem and the subscales sum scores were categorized according to official cut-offs into three groups: noticeable/abnormal, borderline, and normal. Higher scores indicated more mental health problems.

The Screen of Child Anxiety Disorders (SCARED) [27] includes nine items on symptoms of generalized anxiety, which are represented on a 3-point response scale. The German SCARED showed good internal consistency for both parent and self-report versions and proved to be convergently and discriminantly valid [28]. Although Italian adolescents reported higher anxiety scores than their Dutch peers, studies of psychometric properties highlighted that the Italian version of SCARED is a valid screening instrument to rate anxiety symptoms of Italian adolescents [29]. The child version was answered by adolescents. The total sum score was categorized using an international validated cutoff for respondents with and without generalized anxiety symptoms [27].

Seven items from the Center for Epidemiological Studies Depression Scale (CES-DC) [30] were used to assess self-reported depressive symptoms, which showed good internal consistency in the German language. The scale was validated in the Italian language and showed good psychometric properties [31]. The items were presented on a four-point Likert scale. A mean score, with higher values indicating more severe depressive symptoms, was obtained.

With the adopted HBSC symptom checklist [32], eight different psychosomatic problems are assessed. Parents and adolescents were asked how often young people have experienced complaints such as headaches or sleeping problems within the last week. Items were quantified on a 5-point response scale and compared to the COPSY Germany results.

Two items of the PHQ-2 [33] on a 4-point Likert scale (0 to 3) were summarized. A sum score of 3 was used, according to the official classification, as a cutoff for mental disorders. Higher scores indicate higher disorders. PHQ-2 fulfills important psychometric criteria [34,35].

#### 2.2.4. Data Analysis

The data were analyzed using descriptive statistics, using mean (M) and standard deviation (SD) for metric variables and absolute and relative frequencies for nominal- and ordinal-scaled variables. Chi-square tests were performed to compare categorical variables as well as Kendall-tau-b correlation coefficients. A comparison of parents’ and children’s responses was performed only for the subgroup of 11 to 19 years old scholars. Low HRQoL and HBSC subscales were compared using the McNemar test for paired variables. Significance levels of alpha < 0.05 *, alpha < 0.01 **, and alpha < 0.001 *** are reported. Multivariate linear regression analyses were performed for all summed scores to account for sociodemographic and pandemic-specific risk factors. The magnitude of the effects of the independent variables in the regression models was determined by comparing the regression coefficients with the actual SD of the measure. According to Cohen [36], the SD of 0.2 was classified as small, 0.5 as medium, and 0.8 as large effect sizes.

The required sample size was calculated using G-Power 3.1.9.4 (Heinrich-Heine-University, Düsseldorf, Germany) and based on a cutoff for a sample size of alpha = 0.05, a power of 0.9, a small effect of 0.1, and 14 predictors. The minimum sample size required was 243.

## 3. Results

### 3.1. Sociodemographics

From 28 May 2021 to 16 June 2021, 6952 parents of children and adolescents aged between 7 and 19 years participated in the online study providing parent proxy reports, corresponding to a participation rate of at least 18.1%. Of these reports, a total of 5159 questionnaires from families with children aged between 7 and 19 years attending a public school were completed and analyzed to provide baseline information about parents and children in the parent’s report, and 2163 self-reports were gathered from children aged 11 to 19 years (Table 1). In terms of their main characteristics, the study sample and participants were largely consistent with the structure of the corresponding scholar population, according to the annual report of the official statistics institute of South Tyrol [37]. This means, that the study sample is representative of the age and gender of the population of South Tyrolian scholars. About 11.8% of the responding parents were male.

The children’s and adolescents’ mean age was 12.0 ± 3.58 years, and the percentage of male scholars was 49.6%. A migration background was found in 11.6% of the families, and single parenthood was found in 8.6% of the respondents. Parental education was high for 51% of the probands, and most of the responding parents were employed (25.4% full-time, 49% half-time; this inequality results from the high percentage of responding mothers). According to [37,38] in South Tyrol, 11.2% of the scholars had a migration background in 2020/21. Percentages of single parenthood in the South Tyrolean population were not available. Parental education was the only variable that clearly did not represent the educational status of the South Tyrolean population aged from 22 to 64 years. In South Tyrol in this age group, 18.2% of the population is classified to have a low educational standard, 55.7% have a middle standard, and 24% have a high educational standard. The overall occupational status corresponds to a relation of 3:1 (full time: half-time) in the South Tyrolean population. Sociodemographic data of only those families for which both parents and youth aged between 11 and 19 years responded are shown in Supplementary Material Table S1.

### 3.2. Perceived Burden of the Pandemic

The perceived burden of the pandemic was rated by parents as well as adolescents. In general, almost half of the parents (49%) stated that they felt considerably or very burdened by the COVID-19 pandemic, 33% of their children felt considerably/very burdened, and 30% of the parents (35% of parents of adolescents) stated that their child felt considerably/very burdened. The correlation between parents’ assumptions about their children’s burden and their children´s actual feelings of being burdened was 0.473 (*p* < 0.001), and between the parents’ own and their children´s own perceived burden was 0.322 (*p* < 0.001).

The burden on scholars themselves and the parents’ belief of their children’s burden was attributed directly to schools because adjustments to the pandemic were stated as being considerably or very high by 60% and 60%, respectively; the correlation was 0.466 (*p* < 0.001). A higher proportion of parents of all scholars (38%) stated that arguments increased in families with their 11 to 19 years old children and adolescents (26%); the correlation was 0.399 (*p* < 0.001). Seventy-six percent of the parents of all age groups and 60% of the adolescents reported social contact between scholars and friends to be reduced; the respective correlation was 0.376 (*p* < 0.001).

According to 69% of parents (80% of the parents of adolescents) and 83% of adolescents, the respective use of digital media has increased; the respective correlation was 0.407 (*p* < 0.001). Parents’ proxy-report data showed that 32% (36% proxy reports of adolescents) of the scholars used digital media for three hours or more for school and 22% (47% proxy reports of adolescents) for private issues. Thirty-two percent of adolescents reported using digital media for more than three hours for school and 49% for more than three hours for private issues (49%); the correlations were 0.667 and 0.633, respectively (each *p* < 0.001).

The general health conditions of the scholars were reported to be very good or excellent by 80% of the parents and 72% of the adolescents; the correlation was 0.514 (*p* < 0.001).

### 3.3. HRQoL during the Pandemic

**Figure 1:**
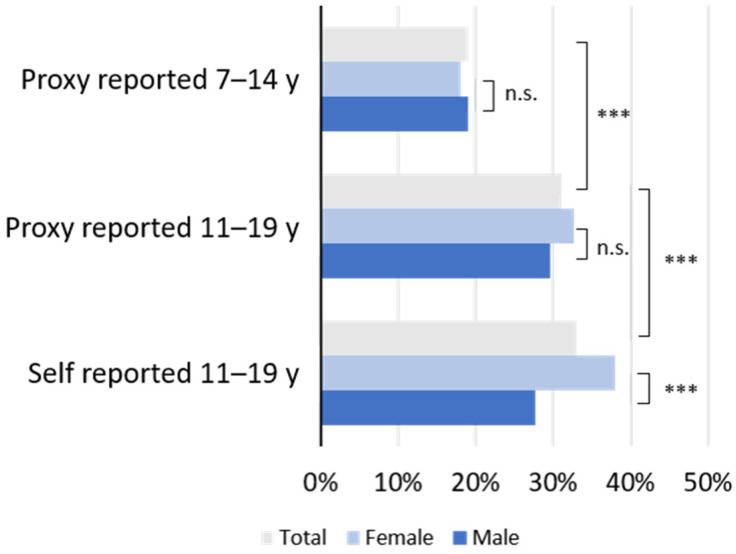
Prevalence of self- and parent (proxy)-reported low HRQoL in children and adolescents of COPSY South Tyrol 2021, stratified by age and gender. The x-axis represents the percentage of scholars categorized “low” by the KIDSCREEN-10 index as described in Materials and Methods. Abbreviations: y, years; n.s., not significant; ***, *p* < 0.001.

Altogether, 27% of the parents’ proxy reports stated a low HRQoL for their children. As shown in Figure 1, a low HRQoL was self-reported by 33% of children and adolescents aged 11 to 19 years, while parents reported a low HRQoL for 31% of their children in this age group. Proxy reports for 11 to 19 years old scholars showed significantly lower HRQoL than proxy reports for 7 to 10 years old scholars. Self-reports of female adolescents returned significantly higher frequencies of low HRQoL than corresponding proxy reports. According to the proxy reports, children aged 7 to 10 years were significantly less affected by low HRQoL than children aged 11–19 years (Figure 1).

The self-reported HRQoL of children and adolescents in COPSY South Tyrol 2021 is compared with data from COPSY Germany in Table 2. In the second year of the pandemic in South Tyrol, low HRQoL of children and adolescents was reported by 33%, which was similar to the percentage of 35% in September–October 2021 in Germany (third wave of the COPSY Germany survey) [18].

### 3.4. Depressive Symptoms during the Pandemic

The prevalence of self-reported depressive symptoms, according to PHQ-2, was 15.3% with a significant difference (*p* < 0.001) between males (10.3%) and girls (20.2%). Younger scholars were significantly less affected than older ones. The results correspond to findings of COPSY Germany obtained in the survey of January 2021 (15.1%) [14], while in the first survey of June 2020 the frequency was 11.3% [13].

### 3.5. Psychological Problems during the Pandemic

The prevalence of parent-reported mental health problems in scholars in South Tyrol 2021 is shown in Table 3. According to the parent’s proxy report, 21.1% of the children and adolescents suffered from mental health problems, demonstrated by a generalized difficulty score indicating borderline or abnormal values. Children aged 7–10 years (22.4%) were not more significantly affected than children aged 11–19 years (20.2%). Comparing COPSY South Tyrol 2021 with COPSY Germany, and especially COPSY Germany 2021 [14] revealed lower prevalence rates in the second year of the pandemic vs. at the end of the first year in that 27% were classified as abnormal/borderline in COPSY Germany 2021 and 21.1% in COPSY South Tyrol 2021.

Emotional problems were reported on the respective subscale at a frequency of 23.6%, which was not different between age groups overall, but more frequent for girls in the adolescent group (girls 30.2% vs. boys 17.2%) and for boys in the age group of 7–10 year (girls 21.4% vs. boys 24.9%), respectively. Conduct problems were reported significantly more often in children in the age group of 7 to 10 years (35.5% vs. 28.2% of 11–19 years old adolescents, and the younger group of boys (39.9%) were affected significantly more often than girls (30.9%). Hyperactivity was reported in 15.1% of the studies, with significantly higher values in younger children and boys. Peer relation problems were found in 28% of the children, with significantly higher values for the group of adolescents (31.2% vs. 23%), with a significant difference in the younger age groups (25.9% vs. 19.9%).

A proportion of 27.1% of the adolescents aged 11 to 19 years suffered from generalized anxiety according to the self-report in COPSY South Tyrol 2021, compared to 24.1% in the COPSY Germany 2020 study of the first wave and 30.1% in the COPSY Germany 2021 third study wave, which is the closest timely related measurement; girls were affected significantly more often than boys (34.6% vs. 19.2%) [18].

### 3.6. Psychosomatic Complaints during the Pandemic

On the HBSC scale, the youth and parents made statements about eight different complaints (Table 4). The frequencies represent cases in which scholars reported complaints at least once in the last week. It was reported that scholars in the age-range 11–19 years felt irritability was most often (65%), followed by feeling nervous (39%), sleeping problems (35%), and feeling low (34%). Psychosomatic complaints were more frequent in girls than boys. Somatic problems in children and adolescents were less frequently reported in the parent’s survey: headache (29%) and stomach aches (28%) were reported most often in the parent’s report, followed by backache (14%) and dizziness (10%).

### 3.7. Associated Risk and Resource Factors

Linear regression models were used for each mental health outcome score, as well as HBSC scores. Linear regression models were calculated for HRQoL from both parents’ proxy reports (M = 47.29; SD = 11.87) and adolescents’ self-reports (M = 48.31 and SD = 10.722); from parent-reported mental health problems (overall SDQ score M = 8.78; SD = 6.01) including the four underscores for emotional problems (M = 2.05; SD = 2.3), conduct problems (M = 2; SD = 1.59), hyperactivity (M = 2.99; SD = 2.36), and peer relational problems (M = 1.75; SD = 1.87); from the generalized anxiety score (M = 5.76; SD = 4.68); for psychosomatic problems using the HBSC score for parent’s proxy reports (M = 36.39; SD = 4.4) and adolescent´s self-reports (M = 34.9; SD = 5.7); and for depressive symptoms from the depression score (M = 11.63; SD = 4.29). Table 5 presents the results.

Younger age was predictive of parents- and adolescents-reported higher HRQoL, higher general SDQ, higher emotional problems, higher conduct problems, higher hyperactivity, lower peer relational problems, lower depressive symptoms, and better self-reported psychosomatic status. Female sex was significantly associated with fewer mental health problems in general (decrease of 34.16% of SD), fewer emotional problems (17.79%), fewer conduct problems (25.2%), less hyperactivity (39.7%), fewer peer relational problems (−16.08%), and higher depressive symptoms (9.01%). The interaction term “age × female gender” was significantly associated with higher emotional and conduct problems and fewer parent and self-reported psychosomatic complaints.

Regarding risk factors, migration background was significantly associated with lower self-reported HRQoL (15%) increased mental health problems (15%), higher hyperactivity (11%), stronger peer problems (20%), and lower parent-reported psychosomatic problems. Single parenthood was significantly associated with increased mental health problems (24%), more emotional problems (15%), stronger conduct problems (19%), stronger peer problems (33%), higher depressive symptoms (23%), and more self-reported psychosomatic symptoms (33%). Low parental education was associated with stronger mental health problems (4%), hyperactivity (11%), anxiety (17%), less depressive symptoms (10%), and better self-reported psychosomatic conditions (11%) while living space without balconies, gardens, and terraces were associated with parent-reported lower HRQoL (36), more mental health problems (39%), stronger emotional problems (38%), and stronger peer problems (33%).

Concerning pandemic-related risk factors, the parental burden due to the pandemic had a significant effect on lower parent-reported HRQoL (11%), lower self-reported HRQoL (13%), more mental health problems (8%), stronger emotional problems (11%) and stronger parent-reported psychosomatic problems (12%) of the scholars. Children’s burden due to changes in school situation was significantly associated with a lower parent’s reported HRQoL (33%) and adolescent’s reported HRQoL (28%), stronger mental health problems (17%), stronger emotional problems (19%), stronger hyperactivity (18%), stronger anxiety (15%), more depressive symptoms (18%), and lower parent-reported psychosomatic conditions (17%). Less contact with friends was associated with a lower parent’s (27%) and children’s reported HRQoL (37%), more mental health problems (8%), stronger peer relational problems (19%), stronger anxiety (16%), and more depressive symptoms (14%). The general burden of children due to the pandemic had a significant effect on all indicators including a lower parent’s reported HRQoL (41%), a lower s adolescent’s reported HRQoL (36%), stronger mental health problems (49%), stronger emotional problems (57%), stronger conduct problems (29%), stronger hyperactivity (34%), stronger peer conduct problems (22%), elevated anxiety (48%), higher depressive symptoms (45%), more parent-reported psychosomatic complaints (44%), and more self-reported psychosomatic complaints (38%).

With regard to resource factors, a lower family climate was significantly affecting all indicators including a lower parent’s reported HRQoL (52%), lower adolescent’s reported HRQoL (60%), stronger mental health problems (63%), stronger emotional problems (57%), stronger conduct problems (62%), stronger hyperactivity (44%), stronger peer relational problems (36%), stronger anxiety (32%), more depressive symptoms (58%), lower parent-reported psychosomatic status (46%), and lower self-reported psychosomatic status (52%). Extended use of digital media affected a parental-reported lower HRQoL (20%), a lower adolescent’s reported HRQoL (10%), stronger mental health problems (12%), stronger emotional problems (11%), stronger conduct problems (14%), and lower parent-reported psychosomatic conditions (7%).

## 4. Discussion

This is the first representative study regarding age, gender, and migration background of South Tyrolean scholars on the HRQoL and mental health of children and adolescents in the entire province of Italy, confirming that children and adolescents in South Tyrol felt significantly burdened by changes in school, reduced contacts, and the pandemic situation overall during the second year of the COVID-19 pandemic. They experienced significantly lower HRQoL and more psychosomatic and mental health problems, especially hyperactivity and peer problems compared with pre-pandemic data from Germany. While younger children seem to be more negatively affected by the pandemic in their mental health problems than older children, low HRQoL and psychosomatic complaints in girls seem to increase during the pandemic by age.

Psychologically distressing experiences of adults living in quarantine during the COVID-19 pandemic in Italy have been documented [39,40]. In an Italian household panel study to track changes in the mental health of survey respondents aged 16 years or older before the onset of COVID-19 and into the first lockdown period, a substantial and statistically significant short-term deterioration in mental health was observed; deterioration was most pronounced among participants aged 16 to 34 years, suggesting that youth may be at increased risk [41].

Reduction in school-related stress during the lockdown may have improved general headaches in children [42]; however, psychosocial health was significantly impaired. Three psychosocial change profiles were identified in a study of youth by latent class analyses: (1) a majority of more than two-thirds experienced a significant increase in psychosocial problems, a smaller subgroup of about one-fifth with increased peer problems only, and a small group of about one-tenth showing no negative change and an increase in prosocial behavior [43]. Neither biological sex nor age emerged as significant moderators of the mental health of children in non-representative and representative studies of the pandemic [14,44]. Other studies reported that missing the school community was a significantly more common cause of psychological problems in girls and the 14–19-year-old group, describing the male gender as a protective factor [45]. Italian adolescents aged 14–19 years were more likely to report increased emotional reactions and anxiety when being a girl, having lost someone from COVID-19, living in a small flat, and not spending time outside; these findings were similar when the Italian survey was repeated in Romania and Croatia [46]. Thus, findings of the roles of age and sex in the psychosocial health of children and adolescents in COPSY South Tyrol 2021 during the second year of the pandemic are in line with some literature reports. However, in COPSY Germany 2020, a well-designed and performed representative survey, no such gender or age effects were found for HRQoL, depressive symptoms, or psychosomatic complaints [14].

In an Italian study, missing school during lockdown was a significantly more common cause of psychological problems in southern Italy than in northern Italy [45]. Studies of northern and southern Italian mothers’ social and didactic parenting previously showed that northern mothers engaged in more social interactions with their infants than did southern mothers [47] suggesting that cultural differences may well play a role in observed geographic differences in mental health results between COPSY South Tyrol 2021 and COPSY Germany 2021. Furthermore, as COPSY South Tyrol 2021 was performed later in the pandemic than COPSY Germany 2020, the duration of the pandemic may differentially affect youths by age and sex. However, additional studies are required to clarify this.

Housing has been identified as a determinant of mental health in COVID-19 lockdowns [48]. Findings suggest that as restriction increases, adolescents’ anxiety and depression become more severe [49]. A worsening of depressive symptoms was more likely observed in participants reporting longer time spent outdoors and more hours of physical activity before the lockdown [50]. In COPSY Germany 2020 wave 2, a reduction of social contacts and less time spent outside was associated with lower HRQoL and higher mental health problems [14]. It is interesting to note that in COPSY South Tyrol 2021, living without balconies, terraces, or gardens appeared to negatively impact mental health more strongly than living space, suggesting that access to outdoor activities may already be beneficial, even if restricted to garden terraces.

Surveys revealed an increase in parenting-related exhaustion due to COVID-19 [51,52,53] and that parents’ distress significantly predicted children’s psychological difficulties [54,55,56]. Although across six different national contexts, including Italy, the experience of the pandemic differed, associations between COVID-19-related family experiences and child adjustment difficulties were similar across countries in nature and magnitude [57]. In a small study performed in the province of Messina, Sicily, children aged 5–16 years reported higher levels of depressive symptoms and event-related anxiety, which increased as children got older, and stress and anxiety in parents were positively correlated with mood depression and anxiety in their children [58]. Similar observations were reported for another non-representative sample aged 7–18 years from different Italian regions [44].

Having a family member or an acquaintance with COVID-19 increased mental health problems, with little difference between northern and southern regions of Italy that were differently hit by the pandemic in the first year [45].

Sleeping difficulties in children and adolescents were frequently reported by both parents and youths (Table 2). The first COVID-19 lockdown affected the health of children and adolescents through an increase in sleep disorders [59]. Home confinement led to a marked delay in sleep timing (i.e., later bedtime and rise time) in children (6–10 years old) and increased emotional, conduct, and hyperactive symptoms that were predicted by the change in sleep quality, boredom, and mothers’ psychological difficulties [60]. Sleep quality appears to be of particular relevance in the interplay between mothers’ and children’s behavioral and psychological factors during COVID-19 [61]. Our finding of more sleeping difficulties in girls than in boys may be related to the increased mental health problems in girls as compared with boys.

In the representative longitudinal COPSY-Germany study, there were larger changes from pre-pandemic to pandemic data, while the changes from wave 1 to wave 2 were rather modest but still indicated that the observed impairments in well-being and mental health seem to be rather stable over the course of the pandemic [14]. Risk factors of particular burden included having fewer social contacts or spending less time outside (due to lockdown measures), being socially disadvantaged, and being children of mentally ill parents, whereas a positive family climate and social support were each associated with better HRQoL and mental health during the pandemic [14]. The first wave of the population-based longitudinal COPSY-Germany study was conducted from 26 May to 10 June 2020, and, after approximately six months, the second wave was conducted between 17 December 2020, and 25 January 2021, and the third wave from 14 September and 11 October 2021. Three and a half months before, COPSY-South Tyrol was performed, where the levels of impairment tended to be lower than those observed in wave 3 of COPSY-Germany. In an international study of sleep timing and quality of adults during the lockdown, the Italian sample underwent significant modifications, especially in unemployed participants; in the Belgian sample, this category suffered less from the restrictions [62], suggesting that involving countries with different types of health and welfare systems, understanding which policy measures have the most effective protective role on physical and mental health is of primary importance. Well-being and mental health in children aged 6–13 years, therefore, will be assessed in the large-scale, international Collaborative Outcomes study on Health and Functioning during Infection Times Children and Adolescents (COH-FIT-C&A—www.coh-fit.com (accessed on 22 April 2022)) project, aiming to identify subgroups of children and adolescents at increased risk of struggling during the pandemic; representative results will be available in the near future [63].

Our study has several strengths and limitations. The strengths of the study include the large population-based sample, the assessment of HRQoL and mental health using established and validated instruments, and the availability of two population-based pre-pandemic reference samples from the first year of the pandemic in Germany. The limitations are similar to those of COPSY Germany and include the possibility of seasonal effects. No allowances were made in the survey for youth with disabilities that may have had difficulty reading and comprehending, which could have excluded youth. Finally, the pandemic burden was assessed with psychometrically unvalidated items, and mental health symptoms were measured using screening questionnaires (no clinical diagnoses were assessed).

Although COPSY studies do not allow any causal conclusions to be drawn about what exactly caused the results found, COPSY South Tyrol suggests that findings from Germany may be generalizable to other regions. Both studies, COPSY South Tyrol and the third wave of COPSY Germany [18] demonstrate lingering impaired HRQoL and mental health for a substantial proportion of children, particularly the at-risk groups, with only slight improvements in mental health. Further research is needed using the same measures, comparable time intervals, designs, and methods, and capturing infection rates and policy responses to the pandemic for both cross- and within-country comparisons.

## 5. Conclusions

Overall, the findings of the population-based COPSY South Tyrol study confirmed observations of increasing mental health problems in children and adolescents during the COVID-19 pandemic at the regional level in Europe. Although it is not yet clear how many additional youths will develop a mental disorder, the development of up-to-date self-harm and suicide statistics to monitor the effects of the current pandemic has already been defined as an urgent priority [64]. Thus, the study results are highly relevant to regional public health and policy. After the first year of the pandemic, lockdown and social distancing measures were already being more carefully balanced with children’s mental health risks. However, the findings nevertheless strongly support the call for the development of targeted and low-threshold health promotion, prevention, and early intervention programs to support children and adolescents severely affected by the pandemic.

## Figures and Tables

**Table 1:** Sociodemographic characteristics of the COPSY South Tyrol 2021 sample.

	Children and Adolescents Aged 7–19 Years (Parent-Report)		Children and Adolescents Aged 11–19 Years (Self-Report)	
	(*n* = 5159)		(*n* = 2163)	
	*n* (%)	M (SD)	*n* (%)	M (SD)
Age		12.0 (3.58)		14.25 (2.35)
7–10 years	2050 (38.9)		–	
11–13 years	1357 (25.7)		939 (43.4)	
14–19 years	1863 (28.4)		1224 (56.6)	
Gender				
Male	2614 (49.6)		1062 (49.1)	
Female	2647 (50.3)		1099 (50.8)	
Other	6 (0.1)		2 (0.1)	
Age of the parent, years		44.4 (6.5)		46.74 (5.5)
Gender of the parents, male	611 (11.8)		248 (11.5)	
Migration background				
No	4080 (88.4)		1763 (88.6)	
Yes	535 (11.6)		226 (11.4)	
Parental education				
Low	1156 (22.4)		524 (24.2)	
Moderate	1303 (26.2)		547 (25.3)	
High	2561 (49.6)		1003 (46.4)	
No information	139 (2.7)		88 (4.1)	
Single parenthood				
No	4641 (88.1)		1945 (89.9)	
Yes	427 (8.1)		180 (8.5)	
Occupational status				
Full-time employed	1306 (25.3)		616 (28.5)	
Part-time employed	2521 (48.9)		1040 (48.1)	
Self-employed	720 (14.0)		289 (13.4)	
Other employment	102 (2.0)		42 (1.9)	
Housewife/househusband	302 (5.9)		130 (6.0)	
Retiree/pensioner	17 (0.3)		12 (0.6)	
On parental leave	109 (2.1)		12 (0.6)	
Unemployed	52 (1.0)		19 (0.9)	
COVID-19				
A family member contracted COVID-19	1511 (30.0)		658 (30.4)	
A relative died of COVID-19	713 (14.2)		333 (15.4)	

Unweighted data. M, mean; SD, standard deviation.

**Table 2:** HRQoL in children and adolescents during the COVID-19 pandemic, stratified by gender (self-report, 11–17 years COPSY Germany [third wave longitudinal study] 2021; self-report, 11–19 years COPSY South Tyrol 2021).

	Survey	Self-Reported			Parent-Reported		
		*n*	Low HRQoL ^1^	Normal/High HRQoL ^1^	*n*	Low HRQoL ^1^	Normal/High HRQoL ^1^
Boys	COPSY Germany September–October 2021	604	194 (32)	410 (68)	n.d.	n.d.	n.d.
	COPSY South Tyrol May–June 2021	923	255 (28)	668 (72)	1031	305 (30)	726 (70)
	*p*-value ^2^		0.060			n.s. ^3^	
Girls	COPSY Germany September–October 2021	568	218 (38)	550 (62)	n.d.	n.d.	n.d.
	COPSY South Tyrol May–June 2021	983	374 (38)	609 (62)	1073	350 (33)	723 (67)
	*p*-value ^2^		<0.001			0.010 ^3^	
Boys and girls	COPSY Germany September–October 2021	1173	412 (35)	761 (65)	n.d.	n.d.	n.d.
	COPSY South Tyrol May–June 2021	1906	629 (33)	1277 (67)	2104	655 (31)	1449 (69)
	*p*-value ^2^		0.227			n.s. ^3^	

^1^ Groups low and normal/high HRQoL according to KIDSCREEN for details, see Methods; data from COPSY Germany 2021 taken with permission from Ref. [18]. Copyright 2022, Ulrike Ravens-Sieberer. ^2^ *p* values resulting from the χ^2^ test comparing the two groups of children and adolescents with low vs. normal/high HRQoL across the COPSY Germany 2021 study, and the COPSY South Tyrol 2021 study of self-reported and proxy-reported HRQoL. ^3^ *p*-value, self-reported vs. proxy-reported. n.d., no data; n.s., not significant.

**Table 3:** Prevalence of parent-reported mental health problems in children and adolescents in the second year of the COVID-19 pandemic in Germany and South Tyrol, stratified by gender and age.

	*n*	Mental Health Problems (Total) ^1^	Emotional Symptoms	Conduct Problems	Hyperactivity	Peer Problems
Boys and girls						
COPSY Germany 2021						
Age 7–17 years	1618	27.0	24.6	17.1	17.3	23.7
Age 7–10 years	402	32.1	27.4	22.6	26.6	19.7
Age 11–17 years	1216	25.3	23.7	15.2	14.2	25.1
*p*-value, age ^2^		0.008	0.145	<0.001	<0.001	0.025
COPSY South Tyrol 2021						
Age 7–19 years	4517	21.1%	23.6%	31.1%	15.1%	28.0%
Age 7–10 years	1779	22.5%	23.2%	35.5%	18.9%	23.0%
Age 11–19 years	2738	20.2%	23.8%	29.2%	12.7%	31.2%
*p*-value, age		0.073	n.s.	<0.001	<0.001	<0.001
Boys						
COPSY Germany 2021						
Age 7–17 years	843	27.6	21.4	17.3	20.1	25.8
Age 7–10 years	221	32.1	27.6	23.1	27.1	22.2
Age 11–17 years	622	26.0	19.2	15.3	17.7	26.2
*p*-value, age		0.082	0.009	0.008	0.003	0.231
COPSY South Tyrol 2021						
Age 7–19 years	2248	23.3%	20.3%	33.2%	19.2%	28.4%
Age 7–10 years	899	27.3%	24.9%	39.9%	23.9%	25.9%
Age 11–19 years	1349	20.6%	17.2%	28.8%	16.1%	31.7%
*p*-value, age		<0.001	<0.001	<0.001	<0.001	0.003
Girls						
COPSY Germany 2021						
Age 7–17 years	773	26.4	28.1	16.8	14.3	22.1
Age 7–10 years	178	32.6	27.4	21.9	26.4	26.3
Age 11–17 years	595	24.7	28.4	15.2	10.6	23.9
*p*-value, age		0.037	0.788	0.034	<0.001	0.033
COPSY South Tyrol 2021						
Age 7–19 years	2269	19.0%	26.8%	28.9%	11.9%	26.6%
Age 7–10 years	880	17.5%	21.4%	30.9%	13.8%	19.9%
Age 11–19 years	1389	19.9%	30.2%	27.6%	9.3%	30.8%
*p*-value, age		n.s	<0.001	0.094	0.001	<0.001
*p*-value, gender						
COPSY Germany 2021						
age 7–17 years		0.370	<0.001	0.071	0.006	0.080
age 7–10 years		0.361	0.795	0.700	0.635	0.268
age 11–17 years		0.724	<0.001	0.079	0.002	0.035
COPSY South Tyrol 2021						
age 7–19 years		<0.001	<0.001	0.005	<0.001	n.s.
age 7–10 years		<0.001	0.042	<0.001	<0.001	0.002
age 11–19 years		n.s.	<0.001	n.s.	<0.001	n.s.

^1^ Groups due to borderline and noticeable/abnormal (grouped into one group) mental health status according to the parent-reported SDQ (see Methods). ^2^ *p*-values resulting from χ^2^-tests comparing groups with borderline and noticeable/abnormal (gathered into one group) status of SDQ by gender and age. n.d., no data; n.s., not significant.

**Table 4:** Subjective health complaints of children and adolescents experienced weekly or more often, according to scholars’ age, gender, and self or proxy assessment.

	Age (Years)	Self (%)		Proxy (%)		*p*-Values ^2^
		Boys	Girls	Boys	Girls	
Headache	7–10	n.d.	n.d.	19.7	22.9	---
	11–19	32.1	47.8	28.3	39.2	***/***
	7–19	n.d.	n.d.	24.9	32.9	---
	*p*-values ^1^	n.d./***/n.d.		n.s./***/***		
Abdominal pain	7–10	n.d.	n.d.	25.5	29.6	---
	11–19	23.7	40.3	21.5	33.8	***/***
	7–19	n.d.	n.d.	23.1	32.1	---
	*p*-values ^1^	n.d./***/n.d.		n.s./***/***		
Backache	7–10	n.d.	n.d.	4.6	6.3	---
	11–19	26.0	32.4	17.7	21.6	***/***
	7–19	n.d.	n.d.	12.5	15.7	---
	*p*-values ^1^	n.d./**/n.d.		n.s./**/*		
Dizziness	7–10	n.d.	n.d.	5.3	5.5	---
	11–19	15.8	25.9	9.8	15.9	**/***
	7–19	n.d.	n.d.	8.0	21.9	---
	*p*-values ^1^	n.d/***/n.d.		n.s/***/***		
Feeling low	7–10	n.d.	n.d.	29.0	22.3	---
	11–19	34.6	48.5	32.3	42.3	*/***
	7–19	n.d.	n.d.	31.0	36.1	---
	*p*-values ^1^	n.d./***/n.d.		***/***/***		
Irritable	7–10	n.d.	n.d.	62.0	60.4	---
	11–19	55.6	66.8	64.6	69.5	n.s/***
	7–19	n.d.	n.d.	63.6	66.0	---
	*p*-values ^1^	n.d./***/n.d.		n.s./*/n.s.		
Nervous	7–10	n.d.	n.d.	37.3	30.8	---
	11–19	39.6	49.6	42.2	42.8	n.s./***
	7–19	n.d.	n.d.	40.3	38.1	---
	*p*-values ^1^	n.d./***/n.d.		**/n.s./n.s		
Sleeping difficulties	7–10	n.d.	n.d.	34.2	38.8	---
	11–19	38.8	45.0	31.2	37.6	n.s./n.s.
	7–19	n.d.	n.d.	32.4	38.1	---
	*p*-values ^1^	n.d./**/n.d.		n.s./**/***		

^1^ Boys vs. girls by age group (7–10 years/11–19 years/7–19 years). ^2^ Boys vs. girls by reporting group (self/proxy). * *p* < 0.05, ** *p* < 0.01, *** *p* < 0.001. Boys 7–10 years; self, n.d.; proxy, *n* = 856. Boys 11–19 years; self, *n* = 926; proxy, *n* = 1303. Boys 7–19 years; self, n.d.; proxy, *n* = 2159. Girls 7–10 years; self, n.d.; proxy, *n* = 845. Girls 11–19 years; self, *n* = 978; proxy, *n* = 1335. Girls 7–19 years; self, n.d.; proxy, *n* = 2180. n.d., no data; n.s., not significant.

**Table 5:** Predictors of HRQoL and mental health in children and adolescents in the second year of the pandemic.

	HRQoL ^1^		Mental Health Problems ^2,3^					Anxiety ^3,4^	Depressive Symptoms ^3,4^	Psychosomatic Complaints	
	Parent-Report ^2^	Self-Report ^4^	Total	Emotional Symptoms	Conduct Problems	Hyperactivity	Peer Problems			Parent-Report ^2^	Self-Report ^4^
Intercept	59.478	63.97	6.687	0.93	1.841	3.065	0.843	2.395	6.98	39.198	39.224
Age	−0.272 ***	−0.825 ***	−0.283 ***	−0.086 ***	−0.073 ***	−0.15 ***	0.027 *	0.048	0.259 ***	−0.04	−0.206 ^4^ **
Female	0.696	−1.857	−2.054 ***	−0.409 *	−0.4 ***	−0.938 ***	−0.301 **	1.123	0.387 ***	0.234	−0.071 ^4^
Female × Age	−0.118	**0.045**	0.186 ***	0.116 ***	0.029 *	0.024	0.017	0.097	0.055	−0.149 ***	−0.217 *
Migration background	−0.884	−1.628 *	0.905 ***	0.154	0.114	0.26 *	0.372 ***	0.235	0.319	−0.516 **	−0.368 ^4^
Single parenthood	−0.951	−1.069	1.434 **	0.342 *	0.305 *	0.159	0.623 ***	0.596	0.982 *	−0.512	−1.85 ^4^ **
Low parental education	−0.024	0.964	0.372	−0.027	0.042	0.253 **	0.103	−0.797 **	−0.429 *	0.022	0.638 ^4^ *
Living space	0.005	0.006	−0.002	0.000	0.000	−0.001	−0.001 *	−0.002	0.000	0.001	0.001 ^4^
Living without balcony, terrace, garden	−4.269 *	−1.039	2.349 *	0.87 *	0.326	0.535	0.613 *	0.745	−0.093	−0.659	−0.779 ^4^
Parents’ burden due to pandemic	−1.284 ***	−1.35 **	0.505 *	0.252 ***	0.034	0.107	0.113	0.414 ^4^	0.285	−0.484 ***	−0.475 ^4^
Children’s burden due to situation at school	−3.828 **	−3.032 ***	1.024 ***	0.428 ***	0.1	0.417 ***	0.081	0.722 ^4,^**	0.751 ^4,^***	−0.696 ***	−0.571 ^4^ *
Less contact with friends	−3.191 **	−6.421 ***	0.502 *	0.081	−0.054	0.128	0.352 ***	0.726 ^4,^**	0.594 ^4,^**	−0.169	0.012 ^4^
Lower family climate	−6.172 **	−3.953 ***	3.788 ***	1.089 ***	0.982 **	1.046 ***	0.672 ***	1.497 ^4,^***	2.469 ^4,^***	−1.872 ***	−2.952 ^4,^***
Extended use of digital media	−2.387 ***	−1.148 *	0.696 ***	0.245 *	0.218 **	0.132	0.108	0.545 ^4^	−0.194 ^4^	−0.274 *	0.362 ^4^
Children’s burden due to pandemic	−4.85 ***	−3.886 ***	2.966 ***	1.313 ***	0.453 **	0.794 ***	0.405 ***	2.231 ^4,^***	1.943 ^4,^***	−1.761 ***	−2.161 ^4,^***
Model fit (Adjusted R^2^)	0.328	0.322	0.295	0.268	0.173	0.193	0.108	0.201	0.242	0.228	0.189

The table indicates regression coefficients for all independent variables, controlling for the other predictors. * *p* < 0.05, ** *p* < 0.01, *** *p* < 0.005. ^1^ Higher value indicates better quality. ^2^ Parent-report 7–19 years. ^3^ Higher value indicates stronger symptoms and complaints. ^4^ Self-report 11–19 year.

## Data Availability

The data presented in this study are available from the corresponding author upon reasonable request.

## Supplementary Materials

The following supporting information can be downloaded from https://www.mdpi.com/article/10.3390/ijerph19095220/s1, Table S1: Sociodemographic characteristics of the COPSY South Tyrol 2021 sample aged 11–17 years.
