# Supplementary material for: Quality of Life and Mental Health in Children and Adolescents after the First Year of the COVID-19 Pandemic: A Large Population-Based Survey in South Tyrol, Italy
Source: Int J Environ Res Public Health. 2022 Apr 25;19(9):5220. doi: 10.3390/ijerph19095220 (PMC9101425; doi:10.3390/ijerph19095220)
Supplement: Supplementary file 1 [file ijerph-19-05220-s001.zip › ijerph-1682932-supplementary.pdf]

Supplementary Materials

Table S1. Sociodemographic characteristics of the COPSY South Tyrol 2021 sample aged 11–17 years

	**Children and adolescents aged 11–19 years (parent-report)**		**Children and adolescents aged 11–19 years (self-report)**	
	**(*n* = 3220)**		**(*n* = 2163)**	
	***n* (%)**	***M* (SD)**	***n* (%)**	***M* (SD)**
Age		14.36(2.39)		14.25(2.36)
11–13 years	1357 (42.1%)		949 (43.95%)	
14–19 years[a]	1863 (57.9%)		1214 (56.05%)	
Gender				
Male	1579 (49%)		1062 (49.1%)	
Female	1637 (50.8%)		1099(50.8%)	
Other	4 (0.1%)		2 (0.1%)	
Age of the parent, years		46.74 (5.6)		46.75 (5.5)
Migration background				
No	2459 (76.4%)		1763 (81.5%)	
Yes	327 (10.2%)		226(10.4%)	
Parental education				
Low	745(23.1%)		524 (24.2%)	
Moderate	817 (25.4%)		547 (25.3%)	
High	1500 (46.6%)		1004 (46.4%)	
No information	158 (4.9%)		88 (4.1%)	
Single parenthood				
No	2806 (87.1%)		1945 (89.9%)	
Yes	287 (8.9%)		180 (8.3%)	
Occupational status				
Full-time employed	904 (28.1%)		616 (28.5%)	
Part-time employed	1450 (45%)		1040 (48.1%)	
Self-employed	461 (14.3%)		289 (13.4%)	
Other employment	59 (1.8%)		42 (1.9%)	
Housewife/househusband	172 (5.3%)		130 (6%)	
Retiree/pensioner	15 (0.5%)		12 (0.6%)	
On parental leave	25 (0.8%)		12 (0.6%)	
Unemployed	31 (1%)		19 (0.9%)	
COVID-19				
A family member contracted COVID-19	957 (29.7%)		658 (30.4%)	
A relative died of COVID-19	464 (14.4%)		333 (15.4%)	

Unweighted data
M mean; *SD* standard deviation
